# Morphological factors differentiating between early lung adenocarcinomas appearing as pure ground-glass nodules measuring ≤10 mm on thin-section computed tomography

**DOI:** 10.1186/s40644-014-0033-x

**Published:** 2014-11-20

**Authors:** Wenjing Xiang, Yanfen Xing, Sen Jiang, Gang Chen, Haixia Mao, Kanchan Labh, Xiaoli Jia, Xiwen Sun

**Affiliations:** 1 Soochow University School of Medicine, Suzhou, Jiangsu, China; 2 Department of Radiology, Shanghai Pulmonary Hospital, Tongji University School of Medicine, 507 Zheng Min Road, Shanghai 200433, China

**Keywords:** Ground glass nodule, Computed tomography, Small lung lesion, Adenocarcinoma, Atypical adenomatous hyperplasia, Adenocarcinoma in situ, Minimally invasive adenocarcinoma

## Abstract

**Background:**

We aimed to compare the morphological features of pure ground-glass nodules (GGNs; diameter, ≤10 mm) on thin-section computed tomography (TSCT) with their histopathological results in order to identify TSCT features differentiating between atypical adenomatous hyperplasia (AAH), adenocarcinoma in situ (AIS) and minimally invasive adenocarcinoma (MIA).

**Methods:**

Between January and December 2013, 205 pure GGNs with a diameter ≤10 mm on TSCT were pathologically confirmed as AAH (40), AIS (95) or MIA (70) lesions. The patients’ age and sex were recorded. The morphological features were evaluated, and maximum diameter and mean CT value were measured for each nodule. *F* test, Pearson χ^2^ test, Fisher exact test and multinomial logistic regression analysis were used to identify factors differentiating between AAH, AIS and MIA. Receiver operating characteristic (ROC) curve analysis was performed for maximum diameter and mean CT value.

**Results:**

*F* test, Pearson χ^2^ test and Fisher exact test revealed that maximum diameter (*P* <0.00001), mean CT value (*P* =0.005), type of interface (*P* =0.005) and presence of air bronchograms (*P* =0.02, n =44) significantly differed among the AAH, AIS and MIA groups. Multinomial logistic regression analysis showed that maximum diameter ≥6.5 mm, a well-defined and coarse interface indicated AIS or MIA rather than AAH; air bronchograms differentiated MIA from AAH; but these parameters did not differentiate between AIS and MIA. A mean CT value less than −520 HU indicated AAH or AIS rather than MIA, but did not differentiate between AAH and AIS.

**Conclusions:**

In the case of pure GGNs measuring ≤10 mm, a maximum diameter ≥6.5 mm, a well-defined and coarse interface indicate AIS or MIA rather than AAH; an air bronchogram can differentiate MIA from AAH. A mean CT value less than −520 HU indicates AAH or AIS rather than MIA.

## Background

According to the new international multidisciplinary classification of lung adenocarcinomas published by the International Association for the Study of Lung Cancer, American Thoracic Society and European Respiratory Society (ERS) [1], adenocarcinomas consist of preinvasive lesions (atypical adenomatous hyperplasia [AAH] and adenocarcinoma in situ [AIS]), minimally invasive adenocarcinomas (MIAs; predominant lepidic growth with ≤5 mm invasion) and invasive adenocarcinomas (predominant lepidic growth with >5 mm invasion). The term bronchioloalveolar carcinoma (BAC) is no longer used. In the new classification, AIS is equivalent to the formerly used term BAC.

Pure ground-glass nodules (GGNs) appear as slight focal opacities on lung windows, and are invisible and contain no solid components on mediastinal windows. Persistent pure GGN is a common computed tomography (CT) finding in a variety of diseases, such as AAH, AIS, MIA, focal fibrosis and organizing pneumonia. It is well-known that GGNs containing a solid portion (mixed GGNs) are a sign of malignancy. Most studies [2]-[4] have investigated the solid components of mixed GGNs; however, few researchers have assessed pure GGNs.

Pure GGNs are occasionally a sign of malignancy [5]. Lee et al. [6] compared the CT features of pure GGNs between patients with preinvasive lesions (AAH or AIS) and those with invasive adenocarcinomas, and found that a lesion size of <10 mm was a determinant for preinvasive lesions. However, in their study [6], lesion sizes were not limited, and they did not compare the CT findings of AAH with those of AIS because both lesions were combined into a single group (preinvasive lesions). Another study [5] has found that in the case of persistent pure GGNs with a diameter ≥10 mm, the size and mass of the nodule are significant factors that differentiate invasive adenocarcinoma from AIS or MIA. However, the differences between AAH, AIS and MIA remain unknown. No large-scale study of pure GGNs has determined the morphological factors that differentiate between AAH, AIS and MIA lesions which appear as pure GGNs with a diameter of ≤10 mm on thin-section CT (TSCT). Thus, the purpose of our study was to compare the morphological CT features of 205 pure GGNs measuring ≤10 mm in diameter with their histopathological results, and identify significant CT features to improve diagnostic accuracy.

## Methods

The data was collected all from a single centre and our institutional review board approved this retrospective study and waived informed consent. However, before undergoing CT, all patients gave written informed consent for the use of their CT data.

### Nodule selection and patients

We retrospectively reviewed the chest CT data of patients who were found to have persistent pure GGNs and obtained a definitive pathological diagnosis between January and December 2013 consecutively. For inclusion in this study, the nodules were required to satisfy the following criteria: (a) pure, unenhanced GGNs detected on TSCT (section thickness, 1 mm or 2 mm), (b) maximum diameter ≤10 mm and (c) pathologically confirmed nodules. Pure GGN was defined as a focal dense lesion with homogeneous attenuation on the lung window, and no solid portion within the lesion on the mediastinal window. On the basis of these criteria, we selected 205 pure GGNs from 191 patients. The patients consisted of 139 women and 52 men with a mean age of 51 years (range, 26–72 years). Population statistics showed pure GGNs were often found in women, the reason is been researched. Thirty-two patients were smokers and the rest were nonsmokers. The GGNs included 40 AAHs, 95 AISs and 70 MIAs. All patients had undergone TSCT because they were suspected to have lung nodules on low-dose CT scans and intended to undergo surgical resection.

### CT

CT scans were obtained using one of two CT scanners (Sensation 64, Siemens Medical Systems, Erlangen, Germany; Brilliance 40, Philips Medical Systems, Best, The Netherlands). The CT parameters were as follows: section thickness, 1 mm on Sensation 64 and 2 mm on Brilliance 40; tube voltage, 120 kVp; tube current, 150–200 mA; lung window width, 1,500 Hounsfield units [HU] and level, −700 HU; and mediastinal window width, 400 HU and level, 20 HU.

### Analysis of CT images

Two board-certified radiologists with 5 and 10 years of experience in chest CT scan interpretation independently reviewed the TSCT images. Both radiologists were blinded to the pathological diagnosis. The patients’ age and sex, maximum diameter, mean CT value and morphological characteristics (including interface, slight lobulation, spiculation, spine-like process, vascular convergence, air bronchograms and pleural retraction) were recorded. The radiologists independently measured the maximum diameter on the transverse lung window image. The diameters they obtained were averaged for each nodule. They also measured the mean CT value of each nodule on the slice that showed the maximum diameter; values were averaged for each nodule. The interface was the border between the tumor and the normal lung tissues, and was classified as ill-defined, well-defined and smooth, and well-defined and coarse [7]. Spiculation was defined as very fine, linear strands (about 1–2 mm long) extending beyond the lesion. Spine-like process was defined as a structure extending from the lesion and having at least one convex border. Vascular convergence and pleural retraction were changes in the adjacent structures. An air bronchogram was defined as a lucency along a regular bronchial wall [8] or a bubble-like lucency (size, 1–2 mm) within the lesion.

When the maximum diameter and mean CT value differed between the two radiologists, the chest CT data were reviewed by the radiologists until they reached a consensus.

### Statistical analysis

Statistical analyses were performed using the SPSS 19.0 software program. Age, sex and CT findings were compared among the AAH, AIS and MIA groups by using the *F* test (age, maximum diameter and mean CT value), Pearson χ^2^ test (sex, interface, slight lobulation, spine-like process, vascular convergence, air bronchograms and pleural retraction) and Fisher exact test (spiculation). *P* values <0.05 indicated significant differences among the three groups. To clarify the differences between any two of the three pathological types, we selected variables with significant differences (*P* <0.05) as independent variables and pathological types as dependent variables for multinomial logistic regression analysis. On the basis of the results obtained from the multinomial logistic regression analysis about the maximum diameter and mean CT value, we regrouped the three groups into two groups. Receiver operating characteristic (ROC) curves were plotted for the maximum diameter and mean CT value to confirm the optimal cut-off that differentiated the two groups.

## Results

The *F* test, Pearson χ^2^ test and Fisher exact test revealed that maximum diameter (*P* <0.00001), mean CT value (*P* =0.005), type of interface (*P* =0.005) and presence of air bronchograms (*P* =0.02) significantly differed among the AAH, AIS and MIA groups (Table 1).

**Table 1:** Variables that significantly differed among the AAH, AIS and MIA groups

**Variable**	**AAH (n = 40)**	**AIS (n = 95)**	**MIA (n = 70)**	***P*** **Value**
Maximum diameter^a^ (mm)	6.4 ± 1.3	7.4 ± 1.6	7.7 ± 1.5	<0.00001
Mean CT value^a^ (HU)	−586 ± 113	−581 ± 101	−532 ± 101	0.005
Interface^b^ (coarse/smooth)	6/34	34/61	32/38	0.005
Air bronchogram^b^ (yes/no)	2/38	24/71	18/52	0.02

*P* <0.05 was considered significant. ^a^Data are presented as mean ± SD. ^b^Data are presented as number of lesions. Age, sex, slight lobulation, spiculation, spine-like process, vascular convergence and pleural retraction are not listed because they did not significantly differ among the three groups.

Since the above tests only identified variables that significantly differed among the three groups and did not identify differences between any two of the three groups, so we performed multinomial logistic regression analysis. This is a statistical method in which one group is a reference group, and the other groups are compared with the reference group to obtain differences between any two groups. This method can be used for three or more categories. For example, using the AAH group as the reference group, we compared the AIS and MIA groups with the AAH group to identify differences between the AAH and AIS groups and between the AAH and MIA groups. We selected the maximum diameter, mean CT value, type of interface and presence of air bronchograms as independent variables and pathological types as dependent variables for multinomial logistic regression analysis. The results of multinomial logistic regression analysis are shown in Table 2.

**Table 2:** Multinomial logistic regression analysis

**Pathology**	**Variable**		**β**	***P*** **Value**	**Odds ratio (OR)**	**95% CI for OR**
The reference group is AAH						
AIS	Maximum diameter	(X1)	4.174	0.005	65	3.594-1173.709
	Interface (coarse)	(X3 = 0)	1.328	0.01	3.8	1.376-10.351
	Interface (smooth)	(X3 = 1)	0			
MIA	Maximum diameter	(X1)	5.466	0.001	236	10.421-5364.593
	Mean CT value	(X2)	0.006	0.01	1.0	1.001-1.010
	Interface (coarse)	(X3 = 0)	1.651	0.002	5.2	1.807-15.034
	Interface (smooth)	(X3 = 1)	0			
	Air bronchogram (yes)	(X4 = 0)	1.708	0.04	5.5	1.099-27.694
	Air bronchogram (no)	(X4 = 1)	0			
The reference group is AIS						
AAH	Maximum diameter	(X1)	−4.174	0.005	0.02	0.001-0.278
	Interface (coarse)	(X3 = 0)	−1.328	0.01	0.3	0.097-0.726
	Interface (smooth)	(X3 = 1)	0			
MIA	Mean CT value	(X2)	0.004	0.006	1.0	1.001-1.008
The reference group is MIA						
AAH	Maximum diameter	(X1)	−5.466	0.001	0.004	0.000-0.096
	Mean CT value	(X2)	−0.006	0.01	1.0	0.990-0.999
	Interface (coarse)	(X3 = 0)	−1.651	0.002	0.2	0.067-0.553
	Interface (smooth)	(X3 = 1)	0			0.036-0.910
	Air bronchogram (yes)	(X4 = 0)	−1.708	0.04	0.2	0.036-0.910
	Air bronchogram (no)	(X4 = 1)	0			
AIS	Mean CT value	(X2)	−0.004	0.006	1.0	0.992-0.999

*P* <0.05 was considered significant. Only variables with significant differences are listed.

### Maximum diameter

**Figure 1:**
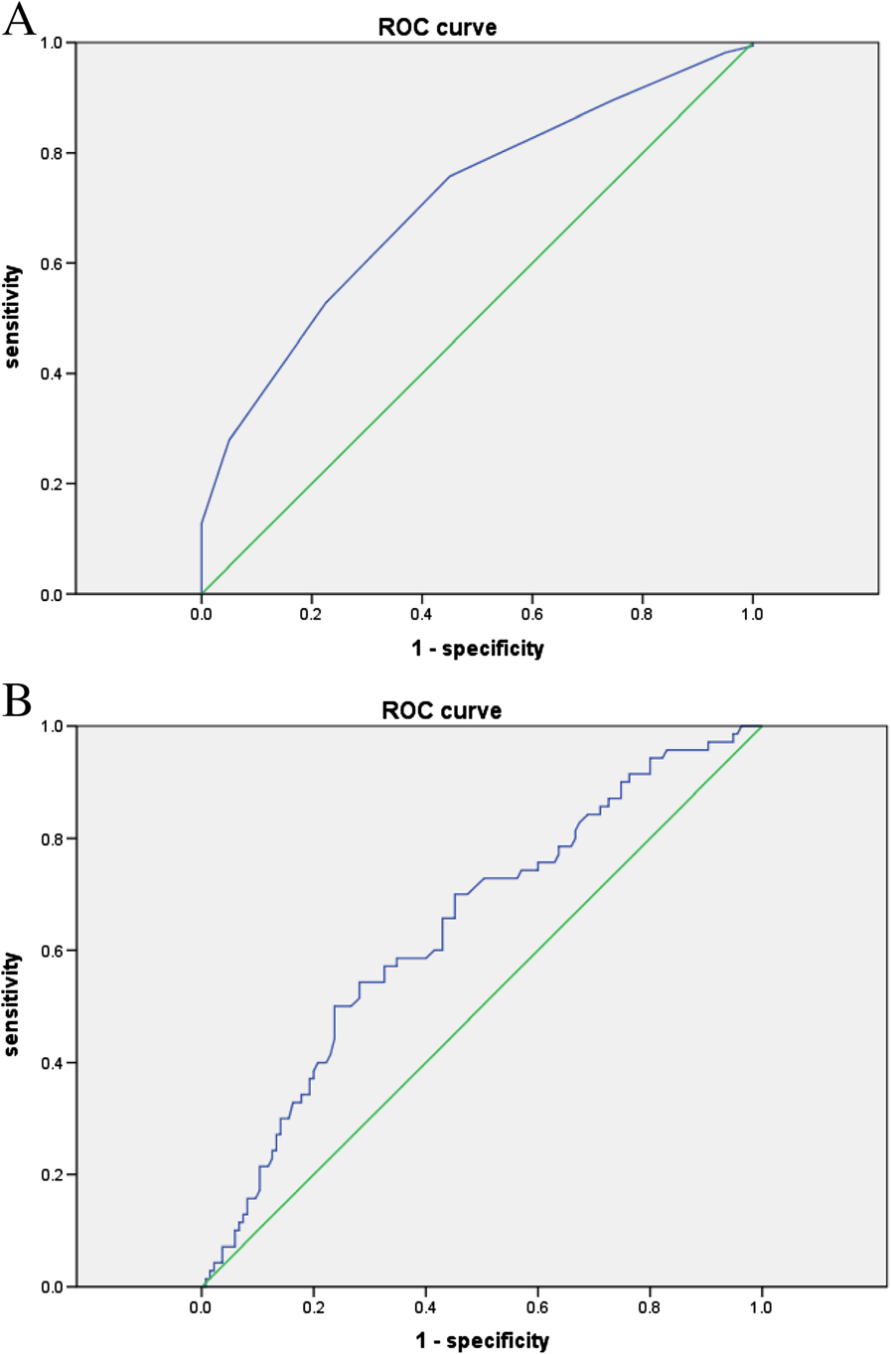
**ROC curves. A**. ROC curve for maximum diameter. **B**. ROC curve for mean CT value.

The maximum diameter significantly differed between the AAH and AIS groups (*P* =0.005, OR =65) and between the AAH and MIA groups (*P* =0.001, OR =236), but not between the AIS and MIA groups. We therefore combined the AIS and MIA groups into a single AIS–MIA group. An ROC curve was plotted between the AAH group and the AIS–MIA group (Figure 1A). The optimal cut-off for distinguishing between AAH and AIS/MIA was 6.5 mm (sensitivity, 75.8%; specificity, 55.0%; area under the curve [AUC], 0.711). Nodules with a maximum diameter <6.5 mm were likely to be AAH, while nodules with a maximum diameter ≥6.5 mm could be AIS or MIA. The odds of a lesion being an AIS or MIA gradually increased with increasing maximum diameter, suggesting that an increase in lesion size increases the possibility of a malignant lesion.

### Mean CT value

Mean CT value significantly differed between the AAH and MIA groups (*P* =0.01, OR =1.0), and the AIS and MIA groups (*P* =0.006, OR =1.0) but not between the AAH and AIS groups. We therefore combined the AAH and AIS groups into a single AAH–AIS group (preinvasive lesions). An ROC curve was plotted between the MIA group and the AAH–AIS group (Figure 1B). The optimal cut-off for distinguishing between AAH/AIS and MIA was −520 HU (sensitivity, 50.0%; specificity, 76.3%; AUC, 0.643). *P* values indicated that mean CT value was significantly different between the AAH and MIA groups, and the AIS and MIA groups. The OR values indicated the mean CT value was not a risk factor that contributed to MIA.

### Interface

We classified the interface as well-defined and smooth, and well-defined and coarse; none of the nodules in our study had ill-defined interfaces. The presence of a well-defined and coarse interface significantly differed between the AAH and AIS groups (*P* =0.01, OR =3.8), and between the AAH and MIA groups (*P* =0.002, OR =5.2), but not between the AIS and MIA groups. An increase in the odds ratio indicated that a well-defined, coarse interface was more likely to be malignant (AIS or MIA) than a well-defined, smooth interface and was rarely related to AAH.

### Air bronchogram

The presence of an air bronchogram significantly differed between the AAH and MIA groups (*P* =0.04, OR =5.5) but not between the AAH and AIS groups, the AIS and MIA groups. The OR value indicated that nodules with air bronchograms were more likely to be MIA than AAH, indicating that air bronchograms were an important malignant feature.

## Discussion

We found that a maximum diameter ≥6.5 mm, a well-defined and coarse interface were helpful to distinguish AIS and MIA from AAH; air bronchograms differentiated MIA from AAH; and a mean CT value less than −520 HU differentiated AAH and AIS from MIA.

In our study, a smaller maximum diameter (<6.5 mm) was associated with AAH, while larger maximum diameters (≥6.5 mm) indicated AIS or MIA. Recently, Lee et al. [6] reported that pure GGNs measuring <10 mm could differentiate preinvasive lesions from invasive adenocarcinomas. Lee et al. [9] reported that persistent pure GGNs measuring >8 mm were likely to be malignant. These reports are consistent with our findings.

Nomori et al. [10] suggested that GGNs with a diameter of ≤10 mm and a histogram CT number exhibiting one sharp peak at less than −650 HU were likely to be AAH rather than BAC. Ikeda et al. [11] reported that AAH can be ruled out when the histogram shows a two-peak pattern, and that the mean CT number is the optimal CT number for differentiating between AIS and adenocarcinoma. We therefore used the mean CT value to evaluate differences between the three pathological types. We found that a mean CT value less than −520 HU indicates AAH or AIS, while a mean CT value greater than or equal to −520 HU indicates MIA. The mean CT value cannot differentiate between AAH and AIS. This is probably because AAH sometimes involves thickened alveolar septa similar to those of AIS, while AIS is sometimes associated with less thick alveolar septa similar to those of AAH. This results in only minor differences in the mean CT values of the two lesions [11]. Cellular and atypical AAH is difficult to differentiate from AIS on histopathological examination [1]. The relatively higher density of MIA may be caused by factors such as tumor fibrosis and interstitial thickening, more cells, less air in the alveoli and alveolar collapse [12].

**Figure 2:**
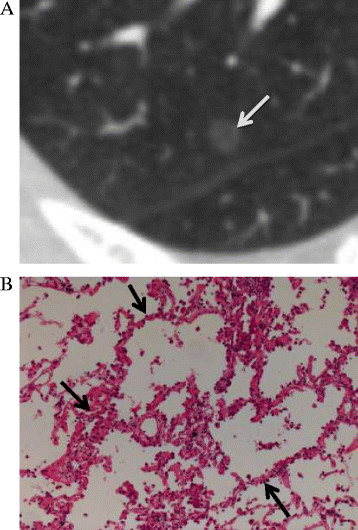
**A 48-year-old woman with atypical adenomatous hyperplasia in the right upper lobe. (A)** A transverse thin-section CT (1 mm thickness) scan shows a pure GGN (arrow) with a maximum diameter of 6 mm, a well-defined, smooth interface and a mean CT value of −723 HU. **(B)** A low-magnification (hematoxylin and eosin, original magnification, ×100) photomicrograph shows atypical, type II pneumocytes and/or Clara cells proliferating inconsecutively along the alveolar walls, no vascular invasion is seen.

**Figure 3:**
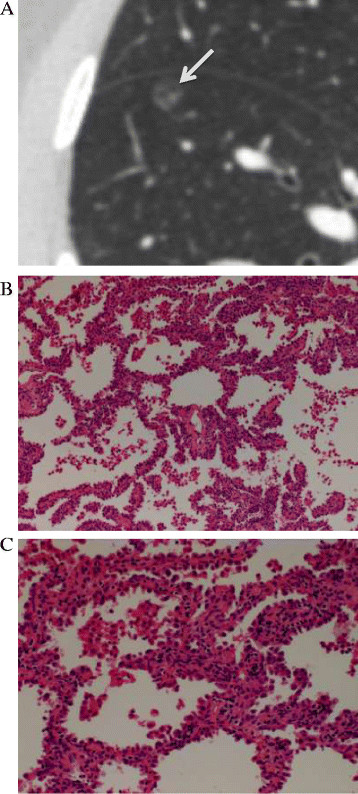
**A 40-year-old woman with adenocarcinoma in situ in the right lower lobe. (A)** Transverse thin-section CT (1 mm thickness) scan shows a round, 7-mm, pure GGN (arrow) with a well-defined, smooth interface and a mean CT value of −678 HU. **(B)** Low-magnification (hematoxylin and eosin; original magnification, ×100) photomicrograph shows atypical pneumocytes proliferating consecutively along the thickened alveolar walls. **(C)** High-magnification (hematoxylin and eosin; original magnification, ×200) photomicrograph shows a spikelike growth of tumor cells.

**Figure 4:**
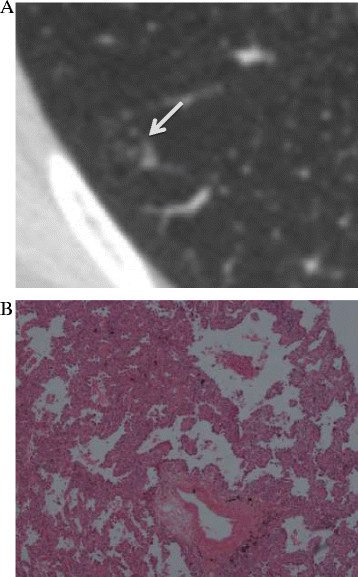
**A 32-year-old woman with minimally invasive adenocarcinoma in the right lower lobe. (A)** Transverse thin-section CT (1 mm thickness) scan shows a 10-mm nodule (arrow) with air bronchograms. Its mean CT value is −755 HU. **(B)** Low-magnification (hematoxylin and eosin; original magnification, ×100) photomicrograph shows atypical pneumocytes proliferating along the thickened alveolar walls, a vascular invasion can be seen.

Interface refers to the border between the tumor and normal lung tissues, and is usually classified into ill-defined, well-defined and smooth, and well-defined and coarse. An ill-defined interface is usually seen in benign lesions, such as inflammation, organizing pneumonia/fibrosis, it’s owing to inflammatory cell infiltration. In most malignant lung nodules, the interface is well-defined and coarse; this is mainly attributable to an infiltrative tumor growth pattern [13],[14]. In a recent study [7] of seven malignant pure GGNs, three had well-defined, smooth interfaces, while four had well-defined, coarse interfaces; there were no ill-defined interfaces. All the nodules included in our study had well-defined (smooth or coarse) interfaces (Figures 2, 3 and 4). We found that a well-defined and coarse interface differentiated AAH from AIS or MIA, but did not differentiate between AIS and MIA.

Air bronchograms have been found to differentiate AIS (*P* =0.007, OR =16.1) from AAH on TSCT images [15]. Kuriyama et al. [16] also reported that air bronchograms can be seen in most malignant tumors. The presence of air bronchograms may be the result of bronchial or bronchiolar invasion by tumor cells and subsequent cartilage or elastic layer changes, airway tortuosity and ectasis [17],[18]. We defined an air bronchogram as a bubble-like lucency (size, 1–2 mm) within the lesion or a lucency along a regular bronchial wall running through the lesion on TSCT (Figure 4). Bronchi were continuous on adjacent TSCT sections. In our study, the Pearson χ^2^ test showed that significantly fewer AAH lesions (5%) were associated with air bronchograms than were AIS (25.3%) or MIA lesions (25.7%). Furthermore, multinomial logistic regression analysis indicated that air bronchograms differentiated MIA from AAH, but could not differentiate between AAH and AIS, AIS and MIA. Histopathological examination of AAH lesions showed atypical, type II pneumocytes and/or Clara cells proliferating along alveolar walls, normal amount of air in the alveoli, no alveolar collapse and no invasion of blood vessels and bronchi (Figure 2). This explains why air bronchograms were rarely seen in AAH lesions. In accordance with our study, Lim et al. [5] have reported that morphological features did not differentiate between AIS and MIA lesions that appeared as pure GGNs.

Our study has several limitations. First, we only selected pure GGNs diagnosed as AAH, AIS or MIA; no cases of invasive adenocarcinomas and organizing pneumonia/fibrosis were included. Second, the mean CT values we measured were the average attenuation values on the transverse slices that showed the maximum diameter of the nodule, not the density of the entire nodule. Last, we used two CT scanners, whose section thicknesses were 1 mm and 2 mm; this difference might have affected the mean CT values and led to inaccuracies in measurements.

The recently published Fleischner Society guidelines [19] recommend the following: (a) CT scanners with 1-mm section thickness should be used for examining GGNs. (b) The average of the maximum and minimum diameters should be taken as the nodule size. (c) Solitary, pure GGNs measuring ≤5 mm do not require follow-up surveillance CT examinations. (d) Solitary, pure GGNs >5 mm require an initial follow-up CT examination in 3 months to determine persistence, followed by yearly surveillance CT examinations for a minimum of 3 years if persistent and unchanged. Surgical resection is recommended for these lesions. Thoracoscopic segmentectomy is considered a better surgical option than thoracoscopic lobectomy for pure GGNs. Lymphatic metastasis rarely occurs after thoracoscopic segmentectomy, which is associated with an excellent prognosis [20],[21].

## Conclusion

In conclusion, in the case of pure GGNs measuring ≤10 mm, a maximum diameter ≥6.5 mm, a well-defined, coarse interface indicate AIS or MIA rather than AAH; air bronchograms can differentiate MIA from AAH. However, these parameters do not differentiate between AIS and MIA. A mean CT value less than −520 HU indicates AAH or AIS rather than MIA, but cannot differentiate between AAH and AIS.

## Abbreviations

AAH: Atypical adenomatous hyperplasia

AIS: Adenocarcinoma in situ

ATS: American Thoracic Society

ERS: European Respiratory Society

GGN: Ground-glass opacity lung nodule

HU: Hounsfield unit

IASLC: International Association for the Study of Lung Cancer

MIA: Minimally invasive adenocarcinoma

TSCT: Thin-section computed tomography

ROC: Receiver operating characteristic

## Competing interests

The authors declare that they have no competing interests.

## Authors’ contributions

Study concepts: XS, WX. Study design: WX, XS, SJ. CT imaging data acquisition: WX. Data analysis and interpretation: WX, XS, SJ. Statistical analysis: WX. Manuscript preparation: WX, YX, HM, KL. Manuscript editing: WX, XS. Tables and figures editing: YX, HM, KL. Manuscript review: XS, SJ, YX. Pathology images: GC and XJ. All authors read and approved the final manuscript.
